# *Burkholderia cenocepacia*–host cell contact controls the transcription activity of the trimeric autotransporter adhesin *BCAM2418* gene

**DOI:** 10.1002/mbo3.998

**Published:** 2020-02-25

**Authors:** Andreia I. Pimenta, Dalila Mil‐Homens, Arsenio M. Fialho

**Affiliations:** ^1^ iBB‐Institute for Bioengineering and Biosciences Instituto Superior Técnico, University of Lisbon Lisbon Portugal; ^2^ Department of Bioengineering Instituto Superior Técnico University of Lisbon Lisbon Portugal

**Keywords:** adhesion to mucins, bacterial adhesion, *BCAM2418* gene, *Burkholderia cenocepacia*–host cell contacts, trimeric autotransporter adhesins

## Abstract

Cell‐to‐cell early contact between pathogens and their host cells is required for the establishment of many infections. Among various surface factors produced by bacteria that allow an organism to become established in a host, the class of adhesins is a primary determinant. *Burkholderia cenocepacia* adheres to the respiratory epithelium of cystic fibrosis patients and causes chronic inflammation and disease. Cell‐to‐cell contacts are promoted by various kinds of adhesins, including trimeric autotransporter adhesins (TAAs). We observed that among the 7 TAA genes found in the *B. cenocepacia* K56‐2 genome, two of them (*BCAM2418* and *BCAS0236*) express higher levels of mRNA following physical contact with host cells. Further analysis revealed that the *B. cenocepacia* K56‐2 *BCAM2418* gene shows an on–off switch after an initial colonization period, exhibits a strong expression dependent on the host cell type, and enhances its function on cell adhesion. Furthermore, our analysis revealed that adhesion to mucin‐coated surfaces dramatically increases the expression levels of *BCAM2418*. Abrogation of mucin O‐glycans turns *BCAM2418* gene expression off and impairs bacterial adherence. Overall, our findings suggest that glycosylated extracellular components of host membrane might be a binding site for *B. cenocepacia* and a signal for the differential expression of the TAA gene *BCAM2418*.

## Graphical abstract

Burkholderia cenocepacia adheres to the respiratory epithelium of cystic fibrosis patients and causes chronic inflammation and disease. Our findings uncover the transcriptional alteration of *BCAM2418* gene induced by the physical contact of the bacterium with bronchial epithelial cells. We found that overexpression of *BCAM2418* gene augmented the bacterial cell adhesion to host cells, and it is dependent on recognition of O‐linked glycans from the host cell membranes.

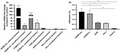

## 1 INTRODUCTION

Bacterial initial contact to host cells has been defined as a crucial step in the overall host–pathogen interaction process (Pizarro‐Cerdá & Cossart, 2006). During the early stages of infection, the ability of the bacteria to sense environmental changes and physical barriers of the host makes the pathogen more prone to alter and adapt its metabolism, regulation, and virulence (Stones & Krachler, 2016). The bacteria could perceive this surface sensing through chemical signals or mechanical forces (Cox, Bavi, & Martinac, 2018). Although poorly understood, it has been described that the sense and adherence of the bacteria to the host could lead to a prompt transcription modulation of a panel of virulence‐associated genes, adhesive molecules, surface antigens, and toxins (Alsharif, Ahmad, Shah, Busby, & Krachler, 2015; Kansal et al., 2013; Katsowich et al., 2017). These physiological alterations can result in a more robust bacterial adhesion that thereby favors colonization and persistence in the host cell (Kansal et al., 2013; Li et al., 2012; Stones & Krachler, 2016).

*Burkholderia cenocepacia* is a human contact‐dependent pathogenic bacterium known for its capacity of adherence and intrinsic interaction with the host, causing severe and persistent opportunistic lung infection in cystic fibrosis (CF) patients (Baldwin et al., 2007; Chiarini, Bevivino, Dalmastri, Tabacchioni, & Visca, 2006). In CF airways, the hypersecretion of mucus (containing water, ions, mucins, and other macromolecules) contributes to the formation of a viscoelastic material that facilitates bacterial adhesion and impairs host immune responses (Colomb et al., 2014; Mullen, Callaghan, & McClean, 2010; Xia, Royall, Damera, Sachdev, & Cummings, 2005). Besides this cell surrounding material, the airway epithelial cells contain membrane‐anchored mucins that represent a group of highly O‐glycosylated transmembrane glycoproteins (MUC1, MUC4, and MUC16 as the most representative). These membrane‐tethered glycoproteins have signaling functions and serve as a protective barrier against invading pathogens (Cullen, O'Connor, Drevinek, Schaffer, & McClean, 2017; Dhar & Mcauley, 2019; Kim, 2012). In contrast to that, in some bacteria, it has been described the use of the mucin carbohydrate moieties as receptors for host cell infection, followed by the modulation of virulence genes expression (Ohneck et al., 2018; Navabi et al., 2012). *B. cenocepacia* uses very complex machinery for primary adherence with host cells in which few adhesion factors or appendages have been described (Dennehy et al., 2016; McClean et al., 2016; Mil‐Homens & Fialho, 2011; Sajjan, Wu, Kent, & Forstner, 2000; Saldías, Ortega, & Valvano, 2009). Among those, the subclass of trimeric autotransporter adhesins (TAAs) (Linke, Riess, Autenrieth, Lupas, & Kempf, 2006) deserves particular attention.

TAAs form a large and diverse group of outer membrane proteins widely distributed in Gram‐negative bacteria. They belong to a subfamily of autotransporter proteins and are secreted to the outer surface of the bacteria via the type Vc secretion system. These proteins have a typical trimeric surface modular architecture, composed of three identical monomers, with a C‐terminal anchor and a variable extracellular set of fiber composed of stalk and globular‐like head regions. While the membrane anchor domain is the defining feature of this class of proteins, highly conserved between all the TAA members, head and stalk organization is adaptable and vary among TAAs (Bassler, Hernandez, Hartmann, & Lupas, 2015; Cotter, Surana, & Geme 2005; Łyskowski, Leo, & Goldman, 2011).

*B. cenocepacia* J2315 possess 7 TAA‐encoding genes distributed between chromosome 2 (*bcaA, BCAM0219, 0223, 1115,* and *2418*) and 3 (*BCAS0236* and *0335*) (Mil‐Homens & Fialho, 2011, 2012; Mil‐Homens, Leça, Fernandes, Pinto, & Fialho, 2014). They have been implicated in the binding to a considerable set of molecules, such as host cell receptors, components of the extracellular matrix or even other TAAs (El‐Kirat‐Chatel, Mil‐Homens, Beaussart, Fialho, & Dufrêne, 2013; Mil‐Homens, Pinto, Matos, Arraiano, & Fialho, 2016). Results showed that TAAs are involved in several virulence‐associated features, such as biofilm formation, evasion to host immune system, motility, invasion of host cells, hemagglutination, and induction of inflammation (Mil‐Homens & Fialho, 2011, 2012; Mil‐Homens et al., 2014, 2016).

In this work, we aimed to uncover the relevance of *B. cenocepacia* TAAs in the early stages of infection. In particular, our findings reveal the transcriptional alteration of *BCAM2418* gene induced by the physical contact of the bacterium with bronchial epithelial cells. Moreover, we found that overexpression of *BCAM2418* gene contributes to the bacterial cell adhesion to host cells and is dependent on recognition of O‐linked glycans from the host cell membranes. Overall, this study not only defines the behavior of the TAAs during the step of bacterial adhesion but also provide insights aiming to determine potential targets for therapeutic proposals.

## 2 EXPERIMENTAL PROCEDURES

### 2.1 Bacterial growth conditions

The *B. cenocepacia* clinical isolate K56‐2 is clonally related to the reference strain J2315 (Holden et al., 2009) and was kindly provided by Prof. J. J. LiPuma (University of Michigan, USA). Bacteria were routinely cultured in Luria–Bertani (LB) broth (NZYTech), at 37ºC with orbital agitation (250 rpm).

### 2.2 Human cell lines and cell culture conditions

Two human bronchial epithelial cell lines were used: 16HBE14o‐ cell line, which is healthy lung cells expressing a functional CF transmembrane conductance regulator, and CFBE41o‐ cell line, which is homozygous for the delta F508 mutation corresponding to a CF airway. Both immortalized cell lines were kindly provided by Dr. Gruenert and coworkers (Bruscia et al., 2002; Cozens et al., 1994). Cells were routinely maintained in fibronectin/‐collagen I‐coated flasks in Minimum Essential Medium with Earle's salt (MEM) (Gibco, ThermoFisher) supplemented with 10% fetal bovine serum (FBS) (Lonza), 0.292 g/L L‐Glutamine (Sigma‐Aldrich) and Penicillin/Streptomycin 100 U/ml (Gibco, ThermoFisher).

Human lung carcinoma cell line A549 (ATCC® CCL‐185™) and human cervix adenocarcinoma cell line HeLa (ATCC® CCL‐2™) were grown in Dulbecco's Modified Eagle Medium (DMEM) (Gibco, ThermoFisher) supplemented with the same components as described above.

The four cell lines were incubated at 37ºC in a humidified atmosphere with 5% CO_2_.

### 2.3 Quantitative assessment of TAAs genes expression upon host cell contact

The transcript levels of TAAs encoding genes *bcaA*, *BCAM0219*, *0223*, *1115*, *2418*, *BCAS0236*, and *0335* were determined by quantitative real‐time PCR (RT‐PCR). RT‐PCR was performed with the 7500 RT‐PCR system (Applied Biosystems) according to the manufacturers' protocols. Total RNA was isolated from adherent *B. cenocepacia* K56‐2 cells (30 min incubation) to the four human cell lines described above.

Bacterial lysis was achieved by enzymatic lysis with lysozyme and proteinase K (Qiagen). Total RNA was purified from a bacterial lysate using RNeasy mini kit (Qiagen), according to the manufacturer's protocol. To avoid contamination with genomic DNA, RNA was treated with RNase‐free DNAse digestion kit (Qiagen) in a column during the purification process, for 1 hr at room temperature. To remove the DNA contamination from isolated RNA, overnight DNase (1 ml for 1.5 mg of RNA) treatment was performed at 37ºC followed by inactivation for 5 min at 65ºC. The total RNA concentration was estimated using a UV spectrophotometer (ND‐1000 UV‐Vis, NanoDrop Technologies).

For RT‐PCR experiments, total RNA was converted to cDNA using TaqMan kit (Applied Biosystems) and then analyzed with Power SYBR Green master mix (Applied Biosystems), using primers to amplify TAA‐encoding genes and *SigA* gene (used as an internal control; Table 1). All samples were analyzed in triplicate, and the amount of mRNA detected normalized to control SigA mRNA values. Relative quantification of genes expression was calculated by using the ΔΔCT method (Livak & Schmittgen, 2001).

**Table 1:** List of RT‐PCR primers used in this study

Gene	Primer	Sequence
*SigA*	Forward	5′‐GCCGATGCGTTTCGGTAT‐3′
	Reverse	5′‐GCGTGACGTCGAACTGCTT‐3′
*BCAM0219*	Forward	5′‐TCTCGGGTACGCGATYGAC‐3′
	Reverse	5′‐TGTTATACAGCTGAAACGACCTTACG‐3′
*BCAM0223*	Forward	5′‐GCAATCGGCCGGAACTC‐3′
	Reverse	5′‐TCGTCTATGCCTCGGTCCAT‐3′
*bcaA*	Forward	5′‐TCACGAGGCGAATTGTCAAC‐3′
	Reverse	5′‐GAGACGTTCACGACATCCGTATC‐3′
*BCAM1115*	Forward	5′‐TTGCCGCAGGCGTATCT‐3′
	Reverse	5′‐CCTTCAGCACCCAGTTG‐3′
*BCAM2418*	Forward	5′‐CGCCAATACCTTCGTTCCA‐3′
	Reverse	5′‐CGGGATAGGCATTGGTGTTG‐3′
*BCAS0236*	Forward	5′‐AACGTGAATCAGCTGAATGCG‐3′
	Reverse	5′‐GGTCTGCTGGATCTGCT‐3′
*BCAS0335*	Forward	5′‐CGACGTAGCTGCCGTTCTT‐3′
	Reverse	5′‐ATTTCGTCGACCGCGGTAAC‐3′

### 2.4 Bacterial adhesion to epithelial cells

Adhesion experiments were carried out on 16HBE14o‐, CFBE41o‐, A549, and HeLa cell lines as described previously (Mil‐Homens & Fialho, 2012), with some modifications. Cells were seeded in polystyrene microplates one day before infection at 1 × 10^6^ cells/mL in a supplemented medium. The cells were infected with a multiplicity of infection (MOI) of 50:1. After infection, plates were centrifuged at 700 *g* for 5 min. The infected monolayers were incubated for a different time periods (15, 30, 120, 180, and 300 min) at 37°C in an atmosphere containing 5% CO_2_. After incubation, each well was washed three times with PBS. For adhesion determination, the host cells were lysed by incubation with lysis buffer (10 mM EDTA, 0.25% Triton X‐100) for 30 min at room temperature. The adhered bacteria were quantified by plating serial dilutions of the cell lysates. Results are expressed as a percentage of adhesion relatively to the initial bacterial dose applied. For further expression assays, the supernatant containing the nonadherent bacteria was recovered, and each well was washed three times with PBS. Cells and adherent bacteria were harvested by scratching and further resuspension in PBS. All the samples were centrifuged, suspended in RNAprotect Bacteria Reagent (Qiagen), and centrifuged again. The recovered pellets were storage at −80ºC until total RNA extraction and further gene expression assays.

### 2.5 Extracellular digestion of surface‐exposed host cell membrane components

16HBE14o‐ cells were seeded 1 day before infection at 1 × 10^6^ cells/mL. Before infection experiment, cellular monolayers were washed two times with HBS (HEPES buffer saline) and incubated with different digestive enzymes like: pronase E (Sigma‐Aldrich; 3.12 μg/ml in HBS), trypsin (Gibco, ThermoFisher; 6.25 μg/ml in HBS,) for 1hr, O‐glycosidase (New England BioLabs; 2,500 U/well), and PNGase *F* (New England BioLabs; 2,500 U/well) for 6 hr at 37°C CO_2_ incubator. The supernatants with the resulting enzymatic‐produced cellular fragments were recovered and inoculated with 5 × 10^7^ CFU/mL of *B. cenocepacia* K56‐2. The samples were incubated for 30 min at 37ºC. The concentrations of all the enzymes were optimized to guarantee the cellular and bacterial viability throughout the assay. HBS and serum‐free MEM were used as controls.

The treated cellular monolayers were washed three times with HBS, and 1 ml of serum‐free MEM was added to each well. The cells were infected with an MOI of 50:1. After infection, plates were centrifuged at 700 *g* for 5 min. The infected monolayers were incubated for 30 min at 37°C in an atmosphere containing 5% CO_2_. After incubation, the supernatant containing the nonadherent bacteria was recovered, and each well was washed three times with PBS. Cells and adherent bacteria were harvested by scratching and further resuspension in PBS. All the samples were centrifuged, suspended in RNAprotect Bacteria Reagent (Qiagen), and centrifuged again. The recovered pellets were storage at −80ºC until total RNA extraction and further gene expression assays.

### 2.6 Adherence to mucins

Bacterial adherence to mucins (mucin from the porcine stomach, type III, bound sialic acid 0.5%–1.5%, Sigma‐Aldrich) was tested as described before with some modifications (Tomich & Mohr, 2003). Briefly, polystyrene microplates were coated with 1 mg/ml mucins (in PBS) and placed at 4ºC, overnight. Extracellular matrix proteins fibronectin and collagen type I were used as controls (10 µg/ml) (Mil‐Homens, Rocha, & Fialho, 2010). The wells were washed twice with PBS and saturated with a 2% (w/v) bovine serum albumin (BSA) (NZYTech) solution for 1 hr at room temperature. The wells were washed twice with PBS. Approximately 5 × 10^7^ CFU/mL was added to each coated well. The plates were incubated 15, 30 min, 2, 3, or 5 hr at 37ºC and washed 3 times with sterile PBS to remove unbound bacteria. The mucin‐coated plates were also subjected to O‐glycosidase treatment, as described previously. Adhesion to treated and untreated mucin‐coated wells was performed during 2 hr at 37ºC.

For adhesion determination, the wells were treated with 0.5% (v/v) Triton X‐100 solution to desorb the bound bacteria. Plates were incubated for 2 hr at room temperature under orbital agitation. One hundred microliters of the content of each well were removed, diluted in PBS, and plated on LB agar plates. Results are expressed as a percentage of adhesion relatively to the initial bacterial dose applied. For further expression assays, the wells were scratched and the released bacteria suspended in PBS. The samples were centrifuged, suspended in RNAprotect Bacteria Reagent (Qiagen), and centrifuged again. The recovered pellets were storage at −80ºC until total RNA extraction and further gene expression assays.

### 2.7 Statistical analysis

All experiments were performed in a minimum of three independent replicates. Relative comparisons were made between corrected values with ANOVA test for significance. A *p* value < .05 was considered statistically significant.

## 3 RESULTS

### 3.1 *B. cenocepacia* K56‐2 TAAs transcripts are produced at different levels after bacterial adhesion to bronchial epithelial cells

**Figure 1:**
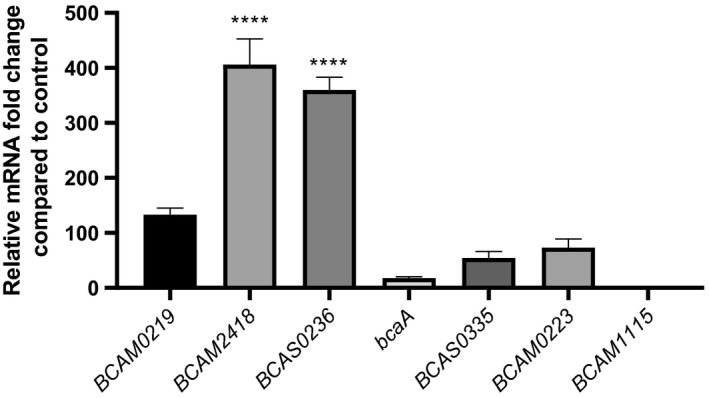
Expression profile of TAA coding genes after adhesion to bronchial epithelial cells. Transcription levels of the 7 *Burkholderia cenocepacia* K56‐2 TAA coding genes were obtained by qRT‐PCR after 30 min of adhesion to 16HBE14o‐ cells. Results were normalized to the expression of the housekeeping *SigA* gene. Expression levels are represented as relative values in comparison to the expression levels in standard LB growth. All the results are from three independent experiments, and bars indicate *SD*. Expression of *BCAM2418* and *BCAS0236* is significantly higher when compared to standard LB growth. (****p < .0001)

We have started this work by analyzing the expression profile of the 7 TAA genes in response to host cell contact (30 min). Thus, we performed quantitative real‐time PCR from RNA samples obtained after *B. cenocepacia* adhesion to 16HBE14o‐ cells. The results of Figure 1 showed a differential expression pattern for all the TAA mRNAs. The expression data are represented in comparison with the basal TAA mRNAs expression obtained from cells that were not subjected to contact with the host cells. The contact with bronchial epithelial cells seems to cause different responses in the TAA‐encoding genes suggesting a differentiated role of the TAAs. Based on the levels of their transcripts expression, TAA genes are divided into two groups. The first one with considerably higher levels of expression (from 100‐ to more than 400‐fold) includes *BCAM0219*, *2418* and *BCAS0236*. A second group with lower expression values includes *bcaA*, *BCAM0223*, *1115*, and *BCAS0335*. *BCAM2418* and *1115* mRNAs are the ones with higher and lower values of expression, respectively, spanning a difference of approximately 400× between them. *BCAM2418* and *BCAS0236* are the only genes that present significantly different levels of expression when compared to basal control (*p* values <.0001; Figure 1). Based on the increased expression of these two genes, we postulate a prominent role of their implicating proteins in the process of bacterial adhesion to host cells. Thus, we decided to pursue this study by characterizing the *BCAM2418* gene, the one that has the higher shift in expression levels.

### 3.2 *BCAM2418* transcriptional levels after adhesion are reliant on the nature of host cells

**Figure 2:**
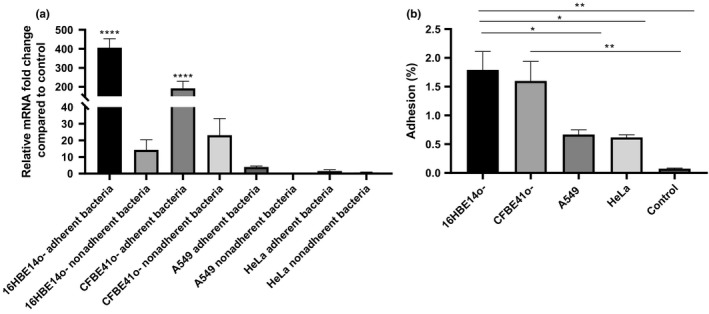
*BCAM2418* transcriptional levels after adhesion to human epithelial cells. (a) Transcriptional levels of the *Burkholderia cenocepacia* K56‐2 *BCAM2418* coding gene were obtained by qRT‐PCR after adhesion to four lines of human epithelial cells: 16HBE14o‐ (bronchial, non‐CF), CFBE41o‐ (bronchial, CF), A549 (lung), and HeLa (cervix). Expression results were also analyzed in nonadherent bacteria samples. Results were normalized to the expression of the housekeeping *SigA* gene. Expression levels are represented as relative values in comparison to the expression levels in standard LB growth. All the results are from three independent experiments, and bars indicate *SD*. Expression of *BCAM2418* after adhesion to 16HBE14o‐ and CFBE41o‐ cell lines is significantly higher when compared to standard LB growth. (****p < .0001). (b) Adherence to 16HBE14o‐, CFBE41o‐, A549, and HeLa cell lines expressed as a percentage of adhesion relatively to the initial bacterial load added to the cells. Adherence to A549 and HeLa cell lines was significantly lower when compared to adherence to both 16HBE14o‐ and CFBE41o‐ cell lines (*p < .05). Adherence to plastic was used as negative control

To determine whether *BCAM2418* expression after cellular contact was a direct response to a specific type of cell, we analyze *BCAM2418* expression patterns after adhesion to a set of human cell lines. Figure 2a represented the results obtained for *BCAM2418* expression after adhesion to HeLa, A549, 16HBE14o‐, and CFBE41o‐ cell lines. It is notorious the difference in the levels of *BCAM2418* mRNAs after adhesion to different cells. The contact with human bronchial epithelial cells, either from a CF or a functional airway, seems to induce a high increase in this gene expression. Adhesion to lung or cervix cell lines does not lead to any alteration in the expression levels.

The expression of *BCAM2418* was also evaluated in nonadherent bacteria, meaning the bacteria recovered in the supernatant after the adhesion assays. The obtained data (Figure 2a) indicate that nonadherent bacteria have lower levels of *BCAM2418* mRNAs when compared to adherent bacteria. These differences are especially evident after adhesion to bronchial epithelial cells. *BCAM2418* expression seems to be induced after bacterial contact with the cellular surface once the nonadherent bacteria do not show the same transcriptional response.

In parallel with the determination of *BCAM2418* gene expression, the adhesion capacity to different host cell lines was also assessed (Figure 2b). The results are expressed in percentage of adhesion relatively to the initial bacterial load added to the cells. The results indicate that adhesion to A549 and HeLa cell lines is inferior when compared to adhesion to the bronchial epithelial cell lines. Bacterial cell adhesion assays to plastic were performed as a negative control. These results seem to follow the same trend of the expression ones, with variations on adhesion percentages coupled to the type of cells used in the assay. Overall data might indicate that *B. cenocepa*cia K56‐2 have a higher adhesion capacity toward bronchial epithelial cells (non‐CF and CF) than to other types of cells.

### 3.3 *BCAM2418* gene expression profile during the early stages of infection

**Figure 3:**
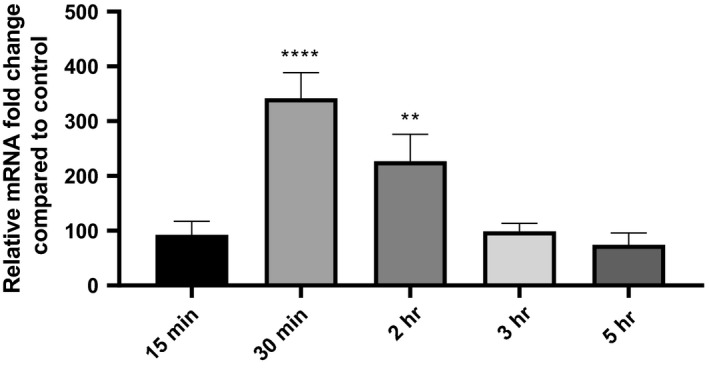
*BCAM2418* transcriptional levels over different times of host cellular contact. Transcription levels of *BCAM2418* coding gene were obtained by qRT‐PCR after different times of adhesion to 16HBE14o‐ cell line—15, 30 min, 2, 3, and 5 hr. Results were normalized to the expression of the housekeeping *SigA* gene. Expression levels are represented as relative values in comparison to the expression levels in standard LB growth. All the results are from three independent experiments, and bars indicate *SD*. Expression of *BCAM2418* after 30 min and 2 hr of adhesion to 16HBE14o‐ cells is significantly higher when compared to standard LB growth. (****p < .0001; **p < .01)

To follow the levels of *BCAM2418* expression after adhesion to 16HBE14o‐ cell line (approximately 400‐fold mRNA expression), we evaluate the transcription of this gene at early and later time points of cellular contact (from 15 min to 5 hr). The results represented in Figure 3 show a timeline pattern of *BCAM2418* expression that reaches a peak after 30 min of adhesion. The same does not occur in the recovered nonadherent bacteria (not shown). The expression of this TAA gene seems to begin to increase soon after the first contact with the host cell (*t* = 15 min), starting to decrease after the adhesion process has taken place (*t* = 2 hr). Moreover, after 5 hr of cellular contact, the levels of *BCAM2418* expression are lower than the ones obtained after the initial 15 min.

### 3.4 Enzymatic treatment of the host cell surface before adhesion cause a decrease in *BCAM2418* transcripts

**Figure 4:**
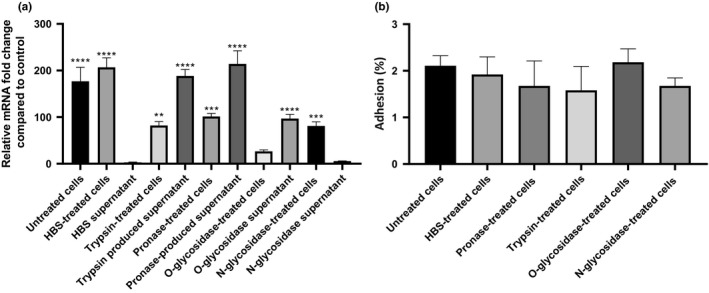
*BCAM2418* transcript levels and *Burkholderia cenocepacia* adhesion after enzymatic treatment of the host cell surface before adhesion. (a) Transcription levels of *B. cenocepacia* K56‐2 *BCAM2418* coding gene were obtained by qRT‐PCR after adhesion to untreated 16HBE14o‐ cell line, HBS‐, pronase‐, trypsin‐, O‐glycosidase‐, and N‐glycosidase‐treated cells. Expression results were also analyzed in bacteria incubated with the supernatant containing the enzymatically produced fragments of the treated cellular surface. Results were normalized to the expression of the housekeeping *SigA* gene. Expression levels are represented as relative values in comparison to the expression levels in standard LB growth. All the results are from three independent experiments; bars indicate *SD*. Expression of *BCAM2418* after adhesion to untreated 16HBE14o‐ cells and to HBS control is significantly higher when compared to standard LB growth. (****p < .0001). *BCAM2418* transcription levels after bacterial incubation with pronase, trypsin, and O‐glycosidase produced supernatant are significantly higher when compared to standard LB growth (****p < .0001). (b) Adherence to 16HBE14o‐ cell line, HBS‐, pronase‐, trypsin‐, O‐glycosidase‐, and N‐glycosidase‐treated cells expressed as a percentage of adhesion relatively to the initial bacterial load added to the cells. All the results are from three independent experiments, and bars indicate *SD*. Adherence to treated cells was not significantly different in comparison to untreated ones (*p > .05)

To evaluate the requirement of a native cellular surface as a stimulus to induce *BCAM2418* expression, we proceed with the enzymatic treatment of the host cell surface before the cellular adhesion. As shown in Figure 4a, the treatment with proteases (pronase E or trypsin) or O‐ and N‐glycosidases before the adhesion event causes a significant reduction in the levels of *BCAM2418* expression in comparison with untreated 16HBE14o‐ cells. This shaving strategy based on limited proteolytic digestion of host surface components (proteins and carbohydrates) correlates with a significant decrease (at least two times lower) of *BCAM2418* transcripts. In line with these data, we observed that incubation of *B. cenocepacia* cells with the supernatants containing the surface released components impacts on the *BCAM2418* gene transcription. Interestingly apart from N‐glycosidase‐treated cells, the values of *BCAM2418* expression after bacterial incubation with these supernatants are higher than the ones obtained after the adhesion to the respective treated cellular monolayers (Figure 4a). These results raise the possibility that the carbohydrate‐binding proteins or other glycoconjugates found on the host cell surface may serve as the recognition signals that directly or indirectly control the rate of *BCAM2418* expression. Moreover, the *BCAM2418* expression profile observed seems to be finely tuned according to the glycosidase enzymatic pretreatment of the cells. Thus, we hypothesize that O‐ but not N‐glycans may serve as a host cell signal to enhance the *BCAM2418* gene expression. As a complementary approach, we have also assessed the rate of *B. cenocepacia* K56‐2 adhesion to enzyme‐treated cells. As shown in Figure 4b, although slightly reduced when compared to untreated ones, the proteolytic digestion of host cell membrane constituents did not seem to affect cell adhesion significantly.

### 3.5 Adhesion properties and *BCAM2418* expression‐based comparison of *B. cenocepacia* K56‐2 to mucins and extracellular matrix proteins

**Figure 5:**
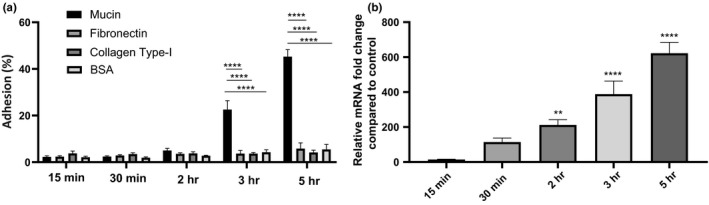
*Burkholderia cenocepacia* adherence to mucins and *BCAM2418* transcript levels over different times of contact. (a) Adherence of *B. cenocepacia* K56‐2 to BSA (control), collagen type I, fibronectin, and mucins during defining timelines—15, 30 min, 2, 3, and 5 hr. Results are expressed as the percentage of adhesion relatively to the initial bacterial load added to the cells. BSA was used as a negative control of the assay. All the results are from three independent experiments, and bars indicate *SD*. The binding capacity to mucins increases over time. The adhesion percentage to mucins is significantly higher after 3h and 5h of contact (****p < .0001). (b) *BCAM2418* transcript levels after adherence to mucins in a defined timeline. Transcription levels of *B. cenocepacia* K56‐2 *BCAM2418* coding gene were obtained by qRT‐PCR after adhesion to mucin coatings during 15, 30 min, 2, 3, and 5 hr. Results were normalized to the expression of the housekeeping *SigA* gene. Expression levels are represented as relative values in comparison to the expression levels in standard LB growth. All the results are from three independent experiments, and bars indicate *SD*. Expression of *BCAM2418* after 2, 3, and 5 hr of mucin adhesion is significantly higher when compared to standard LB growth. (**p < .01; ****p < .0001)

Since one of the primary O‐glycosylated‐type protein of mucus is mucin, we tested the adherence capacity of *B. cenocepacia* K56‐2 to mucins and the impact on *BCAM2418* gene expression profile. Two extracellular matrices (ECM) proteins, namely fibronectin and collagen type I, were used as controls. As shown in Figure 5a, the *B. cenocepacia* K56‐2 exhibited significantly higher binding to mucins when compared to ECM proteins. After 3 and 5 hr of mucin contact, the rate of adhesion reaches approximately 20 and 50%, respectively. In contrast, the percentages of adhesion to the ECM proteins are like the ones obtained with the BSA control and relatively constant in all the adhesion times assessed. Interestingly, we also observed that the effect of high cell adhesion on a mucin layer is directly linked with the levels of *BCAM2418* transcripts. The data indicate that the expression levels of this TAA gene significantly increased over time. The longer the bacteria contact with mucins, the higher the amount of *BCAM2418* transcripts produced (Figure 5b). Taken together, the results described above reveal that *B. cenocepacia* K56‐2 can sense the environment through promoting a specific interaction with mucins which thereby triggers the expression of the *BCAM2418* gene.

### 3.6 Enzymatic deglycosylation of mucins is linked to the ablation of *BCAM2418* expression and a significant reduction of bacterial adhesion

**Figure 6:**
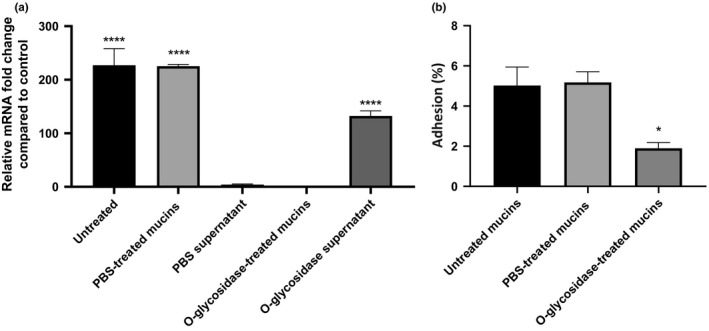
*BCAM2418* transcript levels and *Burkholderia cenocepacia* adhesion after enzymatic treatment of the mucin‐coated surfaces before adhesion. (a) Transcription levels of *B. cenocepacia* K56‐2 *BCAM2418* coding gene were obtained by qRT‐PCR after adhesion to untreated mucin‐coated wells, HBS‐ and O‐glycosidase‐treated mucin‐coated wells. Expression results were also analyzed in bacteria incubated with the supernatant containing the enzymatically produced fragments of the treated surface. Results were normalized to the expression of the housekeeping *SigA* gene. Expression levels are represented as relative values in comparison to the expression levels in standard LB growth. All the results are from three independent experiments; bars indicate *SD*. Expression of *BCAM2418* after adhesion to untreated mucin‐coated wells and HBS control is significantly higher when compared to standard LB growth. (****p < .0001). *BCAM2418* transcription levels after bacterial incubation with O‐glycosidase produced supernatant are significantly higher when compared to standard LB growth (****p < .0001). (b) Adherence to mucin‐coated wells, HBS‐ and O‐glycosidase‐treated wells expressed as a percentage of adhesion relatively to the initial bacterial load added to the cells. All the results are from three independent experiments, and bars indicate *SD*. Adherence to treated mucins was significantly different in comparison to untreated ones (*p < .05)

By placing *B. cenocepacia* K56‐2 cells in contact with a mucin layer, we have observed a significant overexpression of the *BCAM2418* gene and enhancement of the bacterial adhesion. To further investigate this finding, we proceed with the enzymatic deglycosylation of mucins with an O‐glycosidase. As such, we intend to discriminate if the bacterial cell surface recognition is exerted through the binding to the protein itself, their O‐linked glycan cores, or both. As shown in Figure 6a, after the enzymatic deglycosylation of mucins, the *BCAM2418* gene expression was abolished. However, the incubation of *B. cenocepacia* K56‐2 cells with the supernatant containing the O‐linked glycans released from mucins significantly enhances the mRNA expression of *BCAM2418*. Moreover, we also found that the pretreatment of mucins with the O‐glycosidase enzyme can effectively reduce bacterial adhesion (Figure 6b). Taken together, these results indicate that *B. cenocepacia* K56‐2 binds to mucins by mediating recognition to the O‐glycans attached to the external mucin surface. Furthermore, such recognition seems to be mediated, at least in part, via activation of the trimeric autotransporter adhesin gene *BCAM2418*.

## 4 DISCUSSION

*B. cenocepacia* epidemic strains of the ET‐12 lineage are reported to have 7 TAA‐encoding genes (Mil‐Homens & Fialho, 2011; Mil‐Homens et al., 2010). Mil‐Homens and colleagues unveil the multifunctional functions of two *B. cenocepacia* K56‐2 TAAs (BCAM0223 and BcaA) clustered together in chromosome 2 (Mil‐Homens & Fialho, 2011, 2012; Mil‐Homens et al., 2014, 2016). The existence of these surface proteins contributes to the overall pathogenicity of this bacterium (Mil‐Homens & Fialho, 2012; Mil‐Homens et al., 2014). It is known that TAAs can mediate host cell adherence through different types of interactions. These contacts vary from nonspecific events to highly specific bindings between adhesins and host receptors (Ofek, Bayer, & Abraham, 2013). Therefore, TAAs are critical players in defining bacterial tropism to particular host tissue and are determinants of the early stages of infection (Thanassi, 2011). We started this work by assessing the expression levels of the 7 TAA‐encoding genes after 30 min of adherence to host bronchial cells (Figure 1). The TAA genes revealed a different pattern of expression, which may indicate different aptitudes of bacterial adhesion. Among those, *BCAM2418* was the TAA gene with the highest expression after contact with 16HBE14o cells. Little is known about BCAM2418 function for *B. cenocepacia* pathogenicity; *in silico* analyses revealed BCAM2418 as a protein with unique features, like its length, its structural polymorphism and its extensive serine‐rich repeats motifs (Mil‐Homens & Fialho, 2011).

To begin to address the cell specificity to the *BCAM2418* gene response, we compared the bacterial adhesion in a panel of four cell lines. Interestingly, from both bronchial cells tested, 16HBE14o‐ (non‐CF) and CFBE41o‐ (CF), the contact with the non‐CF ones triggers a notable increase in *BCAM2418* expression. These results follow the same trend that the performed adhesion assays, in which the higher adhesion rates are achieved after contact with the non‐CF cells (Figure 2). It seems possible that, at least *in vitro* and under the experimental conditions used, non‐CF cells are preferentially recognized by *B. cenocepacia* K56‐2 when compared to the other studied human cell lines, including the CF cells. Also, direct contact with the host cell is likely a need for *BCAM2418* overexpression, once the nonadherent bacteria do not show increased levels of *BCAM2418* transcription. *BCAM2418* expression appears not to be prompted by any component of the retained supernatant that may content cellular products and secreted molecules. This fact enhances the assumption that is the physical recognition of a cellular target component by *B. cenocepacia* that leads to the increased expression of this TAA mRNA. This hypothesis is reinforced by previous studies that point out the interaction of *B. cenocepacia* and other Bcc species with extracellular molecules of airway cell lines as an essential set point of infection (Mil‐Homens et al., 2016; Pacello, D'Orazio, & Battistoni, 2016).

The host cell proximity seems to be a requirement to stimulate the *BCAM2418* transcription. *BCAM2418* expression follows a differential expression pattern that varies with the time of cellular contact (Figure 3). The highest level of expression was obtained after 30 min of *B. cenocepacia*‐16HBE14o‐ adhesion. The presence of BCAM2418 TAA on the surface of the bacteria could play an essential role in the initial steps of infection. The variation in adhesin genes expression after a particular stimulus is mostly linked to their function and requirement to play a particular role in pathogenesis (Ducret, Hardy, & Brun, 2015; Sheets & St Geme, 2011). Unfortunately, despite many efforts, we failed to obtain a BCAM2418 mutant, and hence, we were unable to perform the mutant phenotypic analysis and determine the impact of this gene product.

Previous studies have shown the specificity of Bcc species to interact with lung epithelial cells (McClean & Callaghan, 2009; Sajjan, Keshavjee, & Forstner, 2004). In this scenario, we aimed to disclosure the cellular component that is recognized by *B. cenocepacia* K56‐2 and prompted an overexpression of the *BCAM2418* gene. It is known that protein or glycoconjugates host receptors mediate binding of Bcc species to the airway host epithelium with bacterial surface components such as flagella, pili, and nonpilus adhesins (Drevinek & Mahenthiralingam, 2010; McClean & Callaghan, 2009; Urban, Goldberg, Forstner, & Sajjan, 2005). In this work, a 16HBE14o‐ cellular monolayer was enzymatically altered using either proteases (trypsin or pronase E) or glycosidases (O‐ or N‐linked). The cell surface shaving significantly reduces the *BCAM2418* transcription in adherent *B. cenocepacia* K56‐2 but does not affect the bacterial adhesion (Figure 4). Moreover, the bacterial incubation with the resulting supernatant fraction seems to restore the signaling and subsequent *BCAM2418* overexpression. It becomes clear the requirement of a physical contact with a protein/O‐linked glycoprotein component of the host cell surface to turn on the *BCAM2418* expression.

These findings led us to hypothesize that membrane‐anchored O‐glycosylated mucins may act as a host cell receptor for *B. cenocepacia* K56‐2 and directly or indirectly leads to the overexpression of the *BCAM2418* gene*.* To further validate this finding, we evaluated bacterial adhesion and *BCAM2418* gene expression on a mucin‐coating surface; ECM‐coated plates were used as control. The effect of mucins, but not the ECM proteins, on bacterial adhesion and further *BCAM2418* overexpression is notorious and increase over time (Figure 5). In this scenario, we were interested in investigating if the adhesion properties and the *BCAM2418* gene expression response to mucin exposure are dictated by the O‐glycans that decorate the mucins or instead by the core protein itself. For these experiments, deglycosylated mucins were used to corroborate that mucin‐associated glycans play a crucial role in bacterial recognition/adhesion, causing in simultaneous the activation of the *BCAM2418* gene (Figure 6).

In the context of CF disease, it is established that *Pseudomonas aeruginosa* and various Bcc species use secretory mucins to adhere to lung epithelial cells (McClean & Callaghan, 2009). Moreover, mucin glycan domains can serve as releasable decoys to prevent infection (Lillehoj et al., 2019; Lindén et al., 2009). Paradoxically, other pathogens have been described to use transmembrane mucins to gain entry into the host cells. This is the case of various *Salmonella enterica* serovars, *Staphylococcus aureus,* and *Helicobacter pylori* where the mucins glycan‐rich domains serve as receptors for infection (Gipson, Spurr‐Michaud, Tisdale, & Menon, 2014; Huang et al., 2016; Li et al., 2019; Navabi et al., 2012; Vesterlund, Karp, Salminen, & Ouwehand, 2006). However, until recently, the structure elucidation of the mucin glycan moieties and their bacterial counterparts involved in the binding events remains difficult to determine. Taken together, our present findings suggest that membrane‐tethered glycosylated mucins exposed on the surface of lung epithelial cells may represent a group of receptors mediating the primary association of *B. cenocepacia* K56‐2 with host cells. We also hypothesize that the TAA BCAM2418, among other putative adhesive factors, operates to promote the initial contact of the bacteria in the infection process.

Comparative DNA sequence analysis of *BCAM2418* genes (structural and promoter regions) of various *B. cenocepacia* isolates revealed that their lengths greatly vary according to the number of repetitive elements. These data support the hypothesis that the *BCAM2418* gene may be subject to phase and antigenic variation during disease. Phase variation permits the on/off switch in expression, while antigenic variation leads to the alteration in the amino acid sequence of extracellular regions of the protein to prevent recognition by the host immune system (Poole et al., 2013). Previous studies have revealed the alteration in TAAs expression in define infection‐linked conditions and environments (Lu et al., 2013; Sheets & St Geme, 2011). The *Haemophilus* cryptic genospecies Cha TAA was found to vary its peptide repeat number gradually during the infection time course. Sheets and St. Geme 3rd found that the expansion of Cha amino acid tandem repeats caused a decrease in Cha binding capability; and also, that this expansion and contraction of the Cha neck motifs could, in theory, balance the bacteria necessity to colonize with the need to disperse in the host or evade the immune system (Sheets, Grass, Miller, & St Geme, 2008; Sheets & St Geme, 2011). *In silico *analyses of BCAM2418 reveal the presence of an extensive number of amino acid repeats that vary in size but seems to maintain an SLST signature (Mil‐Homens & Fialho, 2011). The overexpression of *BCAM2418* might be a mechanism to induce variation in the number of these repeat motifs. This antigenic variation could, in turn, play a similar role to the one reported for Cha TAA. Nevertheless, we could not rule out the alteration in *BCAM2418* expression as a result of an on/off switch. Furthermore, the serine and threonine enrichment of these repetitions may be related to putative O‐linked glycosylation of BCAM2418 extracellular domains (Iwashkiw, Vozza, Kinsella, & Feldman, 2013; Zhou & Wu, 2009). O‐linked glycosylation systems have been described in bacterial systems in the past years, particularly among pathogenic bacteria (Hanuszkiewicz et al., 2014; Vik et al., 2009). The glycosylation of bacterial proteins is usually related to surface and outer membrane proteins. Their sugar enrichment was shown to be linked to adhesive and invasive capacity and protective immunity (Lu, Li, & Shao, 2015; Szymanski & Wren, 2005). Thus, integrated *in* silico and experimental results could bring new insights regarding BCAM2418 importance as an essential virulence factor and a new key player in the earlier steps of *B. cenocepacia* infection.

In summary, we first profiled the expression of the 7 virulence‐associated trimeric autotransporter adhesin (TAA) genes from *B. cenocepacia* K56‐2 during the early stage of bacteria–host cell interaction. Among those, we found that *BCAM2418* gene expression shows an on–off switch and a fine‐tuned control in response to a time frame and a particular host cell environment. We also found that physical contact between the bacterium and the host cell is required to trigger the expression of the *BCAM2418* TAA with the consequent increase of bacterial adhesion. Finally, we hypothesized that the glycosylated extracellular domain of transmembrane mucins might be cell surface receptors used by *B. cenocepacia*. Further research using mucin‐based technologies will contribute to advance our understanding of the mechanisms underlying the early stages of the bacteria–host crosstalk.

## CONFLICT OF INTEREST

None declared.

## AUTHOR CONTRIBUTION

Andreia Pimenta; Conceptualization‐Supporting, Data curation‐Lead, Investigation‐Lead, Methodology‐Lead, Writing original draft‐Lead. Dalila Mil‐Homens; Data curation‐ Supporting, Investigation‐Supporting, Methodology‐Supporting, Supervision‐ Supporting. Arsénio M. Fialho; Conceptualization‐Lead, Data curation‐Supporting, Formal analysis‐Lead, Funding acquisition‐Lead, Project administration‐Lead, Resources‐Lead, supervision‐Lead, Writing review and editing‐Lead.

## ETHICS STATEMENT

None required.

## Data Availability

All data are provided in full in the results section of this paper.
